# Development of a Food Frequency Questionnaire for Assessing Habitual Intake of Free Sugar Among Children in Saudi Arabia

**DOI:** 10.3389/fnut.2021.742737

**Published:** 2022-02-04

**Authors:** Walaa Abdullah Mumena, Hebah Alawi Kutbi

**Affiliations:** ^1^Clinical Nutrition Department, College of Applied Medical Sciences, Taibah University, Madinah, Saudi Arabia; ^2^Clinical Nutrition Department, Faculty of Applied Medical Sciences, King Abdulaziz University, Jeddah, Saudi Arabia

**Keywords:** food frequency questionnaire, free sugar intake, validation, children, Saudi Arabia

## Abstract

The World Health Organization emphasizes the urgency to assess and limit the intake of free sugar (FS) among individuals in order to prevent several non-communicable diseases. However, data regarding intake of FS are lacking in Saudi Arabia and in the Middle East. A reliable valid tool is needed to assess children's habitual intake of FS. Thus, we aimed to develop and validate a food frequency questionnaire (FFQ) that assesses children's habitual intake of FS in Saudi Arabia. In this cross-sectional study, 424 healthy Saudi children ages 6–12 years were included using river sampling method. Sociodemographic data and contact information were collected from mothers using an online survey. Dietary data were collected using 24-h dietary recalls (reference method) and a semi-quantitative FFQ through phone interviews. Items and food groups included in the initial draft of the FFQ were adopted from previous work. Content validity was done to the FFQ with total of 12 food groups and 41 food items. Next, a pilot study was conducted to estimate the sample size needed for the study and to ensure that all items reported in the 24-h dietary recalls were included in the final draft of the FFQ. Finally, data were collected to assess the validity and reliability of the FFQ at the population level. Mean intake of FS assessed by the FFQ was significantly higher than that assessed by the 24-h dietary recalls. Spearman's correlation between total FS assessed by the FFQ and 24-h dietary recall was positively low (*r*_s_ = 0.30, *p* < 0.001). The coefficient alpha indicated an acceptable level of internal consistency (α = 0.74, *p* < 0.001). Test-retest reliability for total FS intake assessed by the FFQ was positively high (*r*_s_ = 0.82). A slight agreement between FS intake assessed by the FFQ and the 24-h dietary recall was seen among the study sample (κ = 0.21, *p* < 0.001). The newly developed FFQ was found to be reasonably valid in assessing children's habitual intake of FS in Saudi Arabia. Validating the instrument among older population in Saudi Arabia is warranted.

## Introduction

The global increase in non-communicable disease epidemics and the documented association with excessive free sugar (FS) consumption had led many countries to establish fiscal policy interventions to limit intake of sugary foods, and it became a global health agenda (1, 2). The impact of excessive free/added sugar intake on health was supported by abundant studies that linked high sugar consumption with overweight (3), dental caries (4), poor dietary quality/intake (5–7), cardiovascular diseases (8), and mortality (9). As an effort to reduce FS consumption among the population, the World Health Organization (WHO) has developed evidence-based guidelines that may further assist policymakers and community programs to reach this goal (10).

The term “free sugar” is used to describe mono- and disaccharides that are naturally present in syrups, honey, and fruit juices, and sugars added to food by consumers or manufacturers, whereas “added sugar” does not include sugar occurring naturally in foods (11, 12). Despite the inconsistences in defining added sugar and FS in research and food labeling, recent recommendations developed by health organizations favors an emphasis on FS due to its impact on health (11). The WHO recommendations include limiting FS intake to <10% and more recently <5% of total energy intake (or <25 grams of FS per day) to prevent excess body weight and tooth decay and to promote overall health (10). This recommendation has been followed by the Saudi Ministry of Health (13). To promote dietary patterns fitting these recommendation, the first step is to assess and monitor FS intake among the populations (10). Assessment of FS intake requires the availability of a validated tool that can be applied in epidemiological studies. Previous studies that attempted to evaluate consumption of FS in Saudi Arabia focused only on certain food items, e.g., sugar sweetened beverages or candies (14–16). Data obtained from such tools cannot provide data regarding total amount of free sugar.

Limiting FS intake among children has been a priority for many intervention programs given that children are one of the most vulnerable groups for excessive sugar intake (17). Another area of concern is that dietary behaviors in childhood may persist into adulthood (18). As such, intervention studies have been devoted to investigate how to promote healthy food choices and dietary habits and limit FS intake effectively among children (19–21). In Saudi Arabia and the Middle East, data regarding intake of FS are lacking. A reliable valid tool is needed to assess children's habitual intake of FS in the region. Thus, the present study aimed to develop a validate a culture-specific food frequency questionnaire (FFQ) to assist in estimating the habitual FS intake among Saudi children.

## Materials and Methods

### Study Design and Population

Saudi healthy children ages 6–12 years were included in this cross-sectional study. Children with food allergy or any chronic disease, siblings of a child whose data were already collected, and children who reside outside of Saudi Arabia were excluded. A pilot sample of 36 children and their mothers was recruited to determine the sample size needed for the present study. Based on the correlation between FS intake from the 24-h dietary recall and from the FFQ (*r* = 0.31), a minimum of 85 children was needed with alpha = 0.05 and power of 80% (22). Ethical clearance to conduct this study was obtained from the ethical committee of the College of Applied Medical Sciences, Taibah University (certificate# 2020/55/202/CLN). Consent form was obtained via the online survey from all mothers before collecting children's data.

### Data Collection

Mothers of children were recruited using river sampling, wherein an online survey was distributed via multiple social media channels, such as WhatsApp, Twitter, and Facebook to collect sociodemographic data (child's age and sex, maternal age and education level, and family income). The online survey was distributed for a duration of 3 months then the data collection was stopped. A text message was sent to all mothers who provided consent for their children to be part of the study to arrange for a phone interview for dietary data collection. The phone interview was conducted by trained dietetic professionals with mothers to collect the dietary data with the presence of the child and the person responsible for child's eating. Dietary data were assessed by 24-h dietary recalls and a semi-quantitative FFQ. During the phone-administered interview, the FFQ was collected followed by the 24-h dietary recall. All mothers received the portion size guide to help them estimate the portion size of foods consumed. The portion size guide included pictures of standardized serving sizes of all food groups (grains, fruits, vegetables, milk and dairy products, meats and alternatives). Pictures of standardized portion sizes of drinks, candies, and sweets were also included in the portion size guide.

### 24-h Dietary Recalls

The 24-h dietary recall was used as a reference method to assess the intake of FS. Single 24-h dietary recall from all children was collected; two additional 24-h dietary recalls (for non-consecutive days) were collected from a subsample of 165 children (38.9%) to adjust for within-person variation. Mothers were requested to remember and report the food intake of children during the past 24 hours. The data obtained from the 24-h dietary recalls were recorded on paper then entered into the Nutritics software https://www.nutritics.com/p/references (version 5.09, Dublin, Ireland) (data source: Nutritics: Middle East/Arabic-Arabic foods/popular Gulf recipes). The Nutritics software provides data concerning content of FS specifically in addition to total sugar and several other nutrients (macro and micronutrients). When amount of FS was not specified for a local food, quantities of FS were estimated based on standardized recipes that were created in the software. The primary investigators trained the data collection team, validated the ingredients of all recipes before inserting them into the software, and monitored the data collection to ensure quality of the data collected. The dietary data were later imported into an Excel sheet in order to calculate the average of the 3 days obtained from the subsample and to prepare the dietary data of all children to be analyzed.

### Development and Validation of the FFQ

The semi-quantitative FFQ was developed to assess the habitual intake of FS. Items of the FFQ were initially adapted from a study that identified the top food sources of added sugar among American children, of which included 17 food groups and 78 food items (17). Content validity was conducted by four experts in the field who provided insight into the FFQ based on diet of the population in Saudi Arabia. Items that are not consumed frequently by Saudis and items that contain low amounts of FS (<5 g of FS in 100 g) were eliminated, including grain drinks, processed meats, and fats/oils. Food items that contain high quantities of FS and were frequently consumed in Saudi Arabia, including chocolate spread, peanut butter, cheesecake, and salad dressings were added into the FFQ. Further, food items that contain FS but were not included in the original FFQ were added to the newly developed FFQ. Similar to the approach used by a Malaysian study to validate a semi-quantitative FFQ (23), we estimated the quantity of FS for each food item included in the FFQ based on standardized portion size to obtain semi-quantitative data. The newly developed semi-quantitative FFQ calculates the quantity of FS from liquid and solid food sources separately as well as the total content of FS (see Table 1). Estimated quantity of FS in each food item was determined using Nutritics software. Portion sizes used in the FFQ were specified based on the standard/mostly used portion sizes of products available in the Saudi market.

**Table 1:** Food groups, food items, and standard portion size included in the FFQ used to assess the habitual intake of free sugar among Saudi children.

	**Food group**	**Food items**	**Standard portion size**
**Liquid food sources of free sugar**			
1	Sweetened	Fruit drink	180 ml
	beverages	Soft drink	330 ml
		Energy drink	250 ml
		Flavored milk	180 ml
		Smoothie	200 ml
**Solid food sources of free sugar**			
2	Ready to eat cereals	Ready to eat cereals: lower sugar ( ≤ 21.2 g/100 g)	30 g
		Ready to eat cereals: higher sugar (>21.2 g/100 g)	30 g
3	Breads and rolls	Yeast breads	35 g
		Rolls and buns	35 g
4	Sweet bakery	Cake	30 g
	products	Fruit pie or cheesecake	30 g
		Cookies	30 g
		Brownies	30 g
		Doughnuts	30 g
		Biscuit	30 g
		Muffins	30 g
		French toast	30 g
		Pancakes/waffles	30 g
		Sweet pastries	30 g
5	Quick breads and	Croissant	30 g
	bread products	Pastries	30 g
6	Candy	Candy containing chocolate	30 g
		Candy not containing chocolate	15 g
7	Other desserts	Ice-cream	125 g
		Popsicle	125 g
		Gelatin	125 g
		Pudding	125 g
8	Sugars	Sugars in tea and coffee	15 g
		Honey	15 g
		Jam	15 g
		Chocolate spread	15 g
		Peanut butter	30 g
		Syrups	15 g
9	Yogurt	Yogurt	175 g
		Flavored yogurt	175 g
10	Mixed dishes	Mixed dishes: pizza or burger, all varieties	140 g
		Mixed dishes: Chinese, all varieties	140 g
11	Condiments and	Ketchup	15 g
	sauces	Salad dressing: ranch, blue cheese, and Italian	30 g
		Salad dressing: french, BBQ, and thousand island	30 g
12	Fruits	Canned fruits	125 g

Next, a pilot sample of 36 children were recruited to ensure that all FS-containing food items reported in the 24-h dietary recalls were listed in the newly developed FFQ. Accordingly, a total of 12 food groups and 41 food items divided into liquid food sources and solid food sources of FS were included in the final version of the FFQ.

The final version of the semi-quantitative FFQ has been later utilized to collect data of children for the present study. During the phone-administered interview, the FFQ was collected followed by the 24-h dietary recall. Firstly, mothers received the portion size guide to help them estimate the portion size of items included in the FFQ. Additionally, a table that includes the frequency response options of the FFQ has been sent to the mothers to ensure accuracy of reporting through showing the response options as follows: per day (once, 2–3 times, 4–5 times, or 6 times or more), per week (once, 2–4 times, or 5–6 times), per month (< once or 1–3 times). Responses to FFQ for each child were recorded on a printed copy of the FFQ then entered into an Excel sheet that calculates the FS content. A separate Excel sheet was used for each child to calculate the total FS intake; the total intake was then inserted in the main Excel sheet that include data for all children. To assess test-retest reliability of the FFQ, data were collected one more time for a subsample of 55 children (13.0%) within 1 month of collecting the first FFQ.

### Statistical Analysis

Descriptive data for continuous variables shown in this study are mean ± standard deviation (SD) or median [interquartile range (IQR)], while data for categorical variables are presented as frequency [percentage (%)]. To validate the semi-quantitative FFQ, Spearman's correlation test was conducted to (1) Investigate the correlation between free FS assessed by FFQ and the reference method (24-h dietary recall); (2) to assess the reproducibility of the newly developed FFQ. Children were grouped into quartiles (same quartile, adjacent quartile, and opposite quartile) to test the agreement in ranking children according to estimated FS intake from both methods. Weighted Kappa statistics was used to assess the interrater reliability across the two dietary collection tools. To evaluate internal consistency of items included in the FFQ, Cronbach's alpha test was used. To assess internal consistency of items included in the FFQ, frequency of consumption of each item was coded between 0 to 8. Responses were coded following the criteria: 0 if the response was less than once a month; 1 if the response was 1–3 times a month; 2 if the response was once a week; 3 if the response was 2–4 times a week; 4 if the response was 5–6 times a week; 5 if the response was once a day; 6 if the response was 2–3 times a day; 7 if response was 4–5 times a day; 8 if response was 6 or more a day. The Bland–Altman plot was used to assess the difference in FS intake reported by 24-h dietary recall and the FFQ compared to the mean intake of the two measures. However, a significant difference was observed between the two methods; therefore Bland–Altman plot was not constructed (24). Paired *t*-test was used to compare the difference between intake of FS assessed by the FFQ and 24-h dietary recall. All tests used in this study were two-tailed with a significant level of 0.05. Statistical Package for Social Sciences (SPSS) version 20 was used to analyze data presented in this study (SPSS, Inc., Chicago, IL, USA).

## Results

The final analysis included data of 424 children after excluding children with chronic diseases and allergies (6.68%, *n* = 36) and children with incomplete dietary data (14.7%, *n* = 79). Characteristics of children and mothers included in this study are described in Table 2. Proportion of children aged 6–7 years was 34.0% (*n* = 144); children aged 8–9 years was 29.7% (*n* = 144); and children aged 10–12 years was 36.3% (*n* = 154). Approximately half of the study sample were girls (*n* = 214). Twenty-five percent of mothers had a high-school degree or less (*n* = 105). Fifty-four percent of mothers reported family income of 10,000 Saudi Riyals (SR) per month or more.

**Table 2:** Characteristics of the study sample (*n* = 424).

	***n***	**%**
**Age group**		
6–7 years	144	34.0
8–9 years	126	29.7
10–12 years	154	36.3
**Sex**		
Boys	210	49.5
Girls	214	50.5
**Maternal age group**		
<30 years	76	17.9
30–40 years	239	56.4
>40 years	109	25.7
**Maternal education level**		
≤ High-school	105	24.8
University	270	63.7
Postgraduate	49	11.6
**Family monthly income**		
< SR 4,000	29	6.80
SR 4,000–10,000	166	39.2
>SR 10,000	229	54.0

Median intake FS assessed by FFQ was 83.5 g/day (58.0–114), while median intake FS assessed by 24-h dietary recall was 42.0 g/day (26.0–60). Mean intake of FS assessed by the FFQ was significantly higher than mean intake of FS assessed by the 24-h dietary recall (94.5 ± 52.8 g/day vs. 44.9 ± 25.7 g/day, respectively, *p* < 0.05) (see Table 3). The correlation between total FS assessed by the FFQ and the 24-h dietary recall was positively low (*r*_s_ = 0.30, *p* < 0.001).

**Table 3:** Mean difference and Spearman's correlation for free sugar intake obtained using food frequency questionnaire and 24-h dietary recall (*n* = 424).

**Free sugar (g)**	**FFQ mean ±SD**	**24-h dietary recall mean ±SD**	**Mean difference**	**% of mean difference**	**Spearman correlation**
All children	94.5 ± 52.8	44.9 ± 25.7	49.6 ± 51.3^a^	110 ± 114	0.30^a^
Boys (*n* = 210)	97.7 ± 54.9	45.7 ± 26.6	52.0 ± 53.4^a^	114 ± 117	0.30^a^
Girls (*n* = 214)	91.4 ± 50.6	44.1 ± 24.8	47.3 ± 49.2^a^	107 ± 112	0.29^a^

a *Significant difference was denoted at confidence level of 95%*.

*Mean difference = Mean FFQ – Mean 24-h dietary recall*.

$\text{Percentage of mean difference}=\frac{\text{Mean FFQ }-\;\text{Mean }24-h\;\text{dietary recall}}{\text{Mean }24-h\;\text{dietary recall}}\times 100$.

Agreement of quartiles between FFQ and 24-h dietary recall is shown in Table 4. Data indicated that 73.6% (*n* = 312) of children were classified into the same and adjacent quartiles, whereas 26.4% (*n* = 112) of children were misclassified. The interrater reliability for the FFQ and the 24-h recalls was found to be κ = 0.21 (95% confidence interval, 0.14–0.28), *p* < 0.001.

**Table 4:** Agreement of quartiles for free sugar intake assessed by the food frequency questionnaire and 24-h dietary recalls (*n* = 424).

	**Agreement of quartiles between FFQ and 24-h dietary recall**				
	**Same quartile**	**Adjacent quartile**	**Opposite quartile**	**Weighted Kappa**	**95% confidence interval**
All children	147 (34.7)	165 (38.9)	112 (26.4)	0.21^a^	0.14–0.28
Boys (*n* = 210)	67 (31.9)	89 (42.4)	54 (25.7)	0.20^a^	0.10–0.29
Girls (*n* = 214)	80 (37.4)	76 (35.5)	58 (27.1)	0.23^a^	0.13–0.33

*Numbers presented in the table are frequencies (%)*.

a *Significant difference was denoted at confidence level of 95%*.

The value of Cronbach's alpha (α = 0.74) suggests good reliability for the FFQ among the study sample. To assess test-retest reliability, Spearman's correlation between FS intake from initial FFQ and repeated FFQ indicated positively high correlation (*r*_s_ = 0.82, *p* < 0.001).

## Discussion

Mean intake of FS assessed by the FFQ was significantly higher than the mean intake of FS assessed by the 24-h dietary recall. Spearman's correlation between the intake of FS assessed by FFQ and 24-h dietary recall was low. The FFQ demonstrated good internal consistency and high level of test-retest reliability.

Mean intake of FS assessed by the FFQ was 110% higher than the mean intake of FS assessed by the 24-h dietary recall. Significant differences in mean FS intake obtained using the FFQ and the 24-h dietary recalls have been also reported previously, where higher estimates were generally obtained by the FFQ (23, 25). Several possible explanations could attribute to the variation between the data obtained from these two dietary collection methods; One is the multiple food items included in the FFQ, which could help in eliminating memory bias and assist in obtaining more accurate estimates of FS intake (23, 26). In fact, foods that are high in FS are most likely consumed between meals, of which can be more difficult to recall compared to foods consumed in regular meals. Besides, underreporting of empty-caloric foods have been previously documented (27, 28). To properly estimate the habitual intake of a particular nutrient, the number of 24-h dietary recalls should be determined based on the variation of nutrient of interest. Otherwise, intake of the nutrient might not accurately reflect its habitual intake. On the other hand, FFQ can be food/nutrient specific, require less resources, and provide data concerning habitual intake of food/nutrient.

Our data collected using FFQ indicated that children consume 94.5 ± 52.8 g of FS per day, estimated approximately as 19 teaspoons. This excessive intake of FS among Saudi children exceeded the AHA by more than three times. In fact, the WHO and other health organizations strongly recommended assessing and monitoring consumption FS intake among populations. However, to our knowledge, studies that evaluated FS intake among children are limited. Several studies suggested high intake of sugary foods and drinks among Saudi children and reported positive correlations with body mass index and waist circumference in these children (29, 30). Given the high prevalence of obesity and associated poor dietary choices/intake among Saudi children (7, 29, 30), a close attention should be paid to dietary intake of these children and associated factors. This is particularly important for children as the current evidence indicates that dietary behaviors of children may last to adulthood (18).

In epidemiologic studies, the usefulness of the FFQ can be considered superior to the 24-h dietary recalls due to its ability to quantify individuals into categories of nutrient intake (31). The FFQ developed in this study demonstrated a significant but slight agreement between the two dietary tools (32), with 34.7% being correctly classified into the same quartile and 38.9% into the adjacent quartile. Spearman correlation between total FS assessed by the FFQ and the 24-h dietary recall indicated a significantly low positive correlation. A review of 227 FFQ validation studies also showed limited correlations between data of the FFQ and the reference methods, ranging between 0.39 and 0.55 (33). The low correlations observed could be explained by the fact that the FFQs typically aim to assess the habitual/usual intake of certain nutrients, reflecting the consumption over a longer duration compared to data obtained from 24-h dietary recalls. The high variability in misreporting dietary data across individuals has possibly attributed to the low correlation between the two dietary assessment tools. The 24-h dietary recall method can be more biased due to the problem of underreporting, specifically FS foods, most likely because food high in FS are consumed between meals (27, 28), whereas the FFQ allows to allocate these foods using direct questions for better estimation of FS intake.

The FFQ developed in this study features with its ability to estimate the habitual intake of FS among Saudi children. The FFQ included culturally specific food items that are commonly consumed by Saudi children. For future research, energy drinks may be eliminated from the FFQ item list due to the very limited consumption among Saudi children, as opposed to American children who were reported to consume energy drinks frequently (17).

This is the first study in Saudi Arabia and the Middle East to develop and validate a tool that specifically assesses the habitual intake of free sugar. The FFQ can further provide data concerning food sources of FS and form of FS (liquid vs. solid). Utilization of the FFQ can offer the opportunity to investigate the associations between FS intake and related diseases. However, validating the FFQ among the different age groups is warranted. Limitations of this study include that the developed FFQ provides dietary data pertaining FS exclusively, whereas information on energy intake and the intake of other nutrients cannot be determined. In addition, the FFQ included food items that contain quantities of FS only. Thus, percentage of FS from total energy intake cannot be calculated.

In conclusion, the newly developed FFQ can be used to estimate the habitual FS intake among children in Saudi Arabia. Future direction may include investigations on possible predictors of excessive FS consumption and relevant food sources. Additionally, validation of the FFQ among different age groups would provide an insight into the relationship between habitual intake of FS and non-communicable diseases.

## Data Availability Statement

The raw data supporting the conclusions of this article will be made available by the authors, without undue reservation.

## Ethics Statement

The studies involving human participants were reviewed and approved by the Ethical Committee of the College of Applied Medical Sciences, Taibah University (certificate# 2020/55/202/CLN). Written informed consent to participate in this study was provided by the participants' legal guardian/next of kin.

## Author Contributions

WM and HK conceived the study, developed the tool, conducted the analyses, interpreted the results, and wrote the paper. All authors critically reviewed the manuscript and approved the final version submitted for publication.

## Conflict of Interest

The authors declare that the research was conducted in the absence of any commercial or financial relationships that could be construed as a potential conflict of interest.

## Publisher's Note

All claims expressed in this article are solely those of the authors and do not necessarily represent those of their affiliated organizations, or those of the publisher, the editors and the reviewers. Any product that may be evaluated in this article, or claim that may be made by its manufacturer, is not guaranteed or endorsed by the publisher.
